# Characterization of NFDQ1 in *Cryptosporidium parvum*

**DOI:** 10.1186/s13071-024-06532-x

**Published:** 2024-10-26

**Authors:** Yangsiqi Ao, Xiaoqing Gong, Jieping Li, Ruiming Zhao, Shujiao Song, Yaqiong Guo, Yaoyu Feng, Lihua Xiao, Rui Xu, Na Li

**Affiliations:** State Key Laboratory for Animal Disease Control and Prevention Center for Emerging and Zoonotic Diseases, College of Veterinary Medicine, South China Agricultural University, Guangzhou, 510642 China

**Keywords:** *Cryptosporidium parvum*, CRISPR/Cas9, NFDQ

## Abstract

**Background:**

*Cryptosporidium* spp. are important zoonotic parasites that can cause moderate to severe diarrhea in humans and animals. Among the three *Cryptosporidium* species infecting the intestines of calves, *Cryptosporidium parvum* has a broad host range and causes severe diarrhea in calves, while *Cryptosporidium bovis* and *Cryptosporidium ryanae* mainly infect calves without obvious clinical symptoms. Comparative genomic analysis revealed differences in the copy number of genes encoding the nonfinancial disclosure quality (NFDQ) secretory protein family among the three species, suggesting that this protein family may be associated with the host range or pathogenicity of *Cryptosporidium* spp. To understand the function of cgd8_10 encoded NFDQ1, tagged and knockout strains were constructed and characterized in this study.

**Methods:**

To determine the localization of NFDQ1, we used clustered regularly interspaced short palindromic repeats (CRISPR)/CRISPR-associated protein 9 (Cas9) technology to tag the C-terminus of NFDQ1 with three hemagglutinin epitopes (3 × HA). The tagged strain was constructed, and the genomic insertion was confirmed by polymerase chain reaction (PCR). Immunofluorescence assays were performed to observe the localization of NFDQ1 both in extracellular sporozoites and at various intracellular developmental stages. Immunoelectron microscopy was used to study the ultrastructural localization of NFDQ1. Then, the *ΔNFDQ1* strain was generated by CRISPR/Cas9 and the in vitro growth assay on HCT-8 cells was used to analyze of phenotypic changes after knockout *NFDQ1* in parasites.

**Results:**

The *NFDQ1* tagging and knockout stains were successfully constructed by CRISPR/Cas9 technology and the insertions of transgenic strains were validated by PCR. The expression of NFDQ1 was validated in parasite by western blot. Immunofluorescence and immune-electron microscopy assay showed that NFDQ1 expressed in both asexual and sexual stages of *C*. *parvum*, where it was localized to the cytoplasm of the parasite. Upon ablation of *NFDQ1*, the *ΔNFDQ1* strain showed an apparent growth retardation during sexual replication in vitro.

**Conclusions:**

NFDQ1 is a cytoplasmic protein without specific localization to secretory organelles, and it may participate in *C*. *parvum* growth during sexual reproduction. Future study should determine the role of NFDQ1 following *C*. *parvum* infection in vivo.

**Graphical Abstract:**

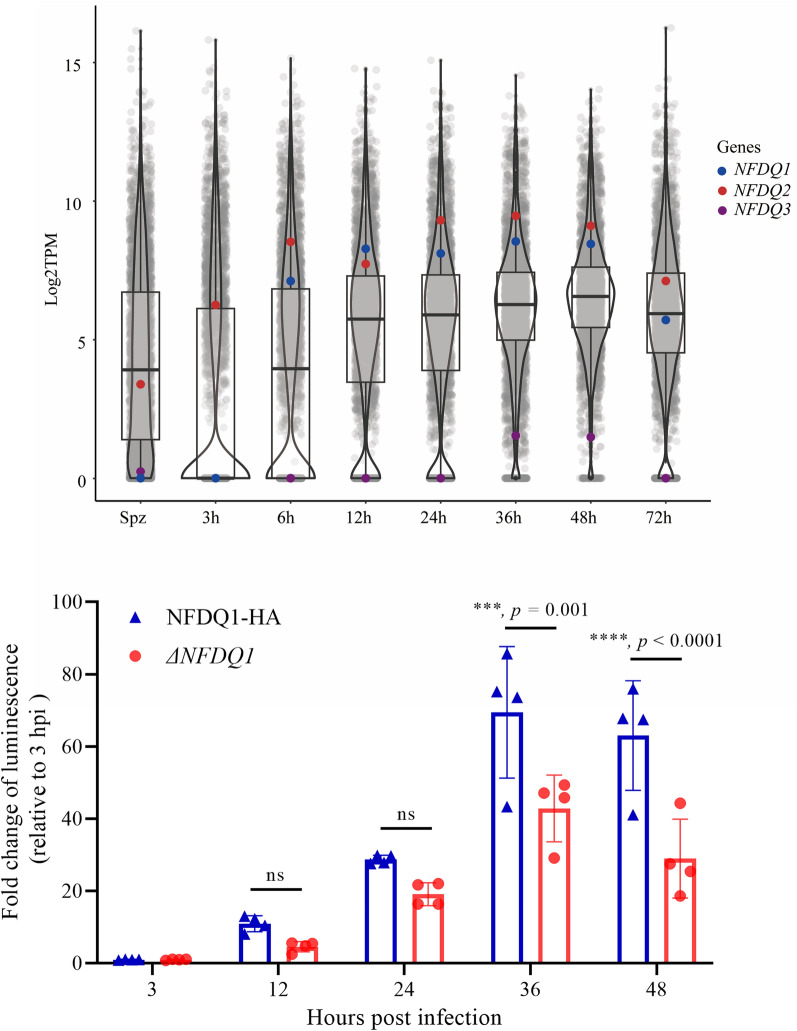

**Supplementary Information:**

The online version contains supplementary material available at 10.1186/s13071-024-06532-x.

## Background

*Cryptosporidium* spp. are important zoonotic parasites that can cause moderate to severe diarrhea in humans and various animals [1]. Almost 7.6% people worldwide are infected with this diarrheal pathogen, and cryptosporidiosis has become the second major disease of infantile diarrhea in developing countries [2, 3]. Clinical symptoms associated with cryptosporidiosis include diarrhea, nausea, vomiting, headache, and abdominal cramps [4]. At present, there is no drug that can effectively treat cryptosporidiosis, and nitazoxanide, the only drug approved by the US Food and Drug Administration (FDA) for the treatment of cryptosporidiosis, is not effective in immunocompromised individuals [5].

To date, nearly fifty *Cryptosporidium* species have been found worldwide [6–9]. Among them, *C*. *parvum* has attracted significant attention owing to its high virulence and high prevalence in humans and animals [10, 11]. In addition to *C*. *parvum*, *C*. *bovis*, *C*. *ryanae*, and *C*. *andersoni* are the major species of *Cryptosporidium* infecting calves [12]. Among them, *C*. *parvum* is the most prevalent in preweaned calves, causing moderate to severe diarrhea or even death [13]. *C*. *bovis* and *C*. *ryanae* are frequently associated with infection of the small intestines of post-weaned calves with no overt clinical symptoms [14–16]. In contrast, *C*. *andersoni* infects the abomasum of adult cattle, resulting in stunted growth, weight loss, and decreased milk production [17].

The highly polymorphic multigene family in the subtelomeric regions is closely associated with the virulence and host range of *Cryptosporidium* [12, 18]. The genomes of *C*. *hominis*, which has a narrow host range, and *C*. *parvum*, which has a broad host range, are highly similar at nearly 97% [19, 20]. The genomic differences between the two species are primarily concentrated in the subtelomeric regions, specifically in the multicopy gene families, including the MEDLE protein family, insulinase-like proteases (INS), and mucin-type glycoproteins. *C*. *parvum* has six copies of the MEDLE protein, while *C*. *hominis* has only one copy [19]. Similarly, the subtelomeric regions of chromosomes 5 and 6 in *C*. *hominis* lack the INS19 and INS20 genes, which are present in *C*. *parvum* [21]. Additionally, *C*. *tyzzeri* infecting a narrow range of hosts also shows differences in the copy numbers of the MEDLE and INS protein families. Previous studies have found that the infection patterns of *C*. *tyzzeri* subtypes IXa and IXb in mice differ significantly, which may be related to phenotypic variations in mucin-type glycoproteins [22].

Comparative genomics studies have demonstrated that the sequence polymorphisms of NFDQ proteins may result in phenotypic differences among *Cryptosporidium* species, including variations in pathogen growth rate, infection duration, and intensity [18]. Each member of the NFDQ proteins has a conserved amino acid sequence beginning with NF and ending with DQ. The three subtellomeric genes (cgd6_5500, cgd6g5500, cgd8_10) of the NFDQ family had different copy numbers in *C*. *parvum*, *C*. *bovis* and *C*. *ryanae* [18]. On the basis of the analysis, the *C*. *bovis* and *C*. *ryanae* genome encodes only the cgd6_5500 gene, lacking the orthologous genes of cgd6g5500 and cgd8_10 [18]. Among the NFDQ family, cgd8_10 is one of the highly polymorphic genes that has been identified in *C*. *parvum*, while few studies have been conducted on the biological properties [23].

In this study, we focused on *NFDQ1* encoded by cgd8_10, which is only present in *C*. *parvum* but absent in *C*. *bovis* and *C*. *ryanae*. We demonstrated that NFDQ1 expressed in both asexual and sexual stages of *C*. *parvum* and localized to the cytoplasm of the parasite. We then used genetic manipulation tools to disrupt the *NFDQ1* gene in *C*. *parvum* and showed that NFDQ1 is involved in parasite growth in vitro.

## Methods

### Animal studies and ethical approval

Animal studies on mice were approved by the Laboratory Animal Research Center of Guangdong Province and the South China Agricultural University (approval number: 2022c002). *Ifnγ*^−/−^ mice (referred to as GKO) (stock no.: 002287) were purchased from the Institute of Laboratory Animal Science, Chinese Academy of Medical Sciences and bred in-house at the Laboratory Animal Center of South China Agricultural University and were separated by sex after weaning. Male and female mice aged 3–5 weeks were used for infection experiments.

### Oocyst preparation and excystation

*Cryptosporidium parvum* IIdA20G1-HLJ strain was isolated from dairy calves in Heilongjiang (HLJ) Province, China, and stored at 4 ℃ in phosphate-buffered saline (PBS) plus 100 U/mL penicillin (Gibco, Grand Island, NY, USA), 100 μg/mL streptomycin (Gibco), and 0.25 μg/mL amphotericin B (Gibco) [24]. Prior to infection, *C*. *parvum* oocysts were treated in 1.3% sodium hypochlorite for 10 min on ice and then washed three times in PBS. To obtain free sporozoites, the sodium hypochlorite-treated oocysts were resuspended in PBS containing 0.75% sodium taurodeoxycholate (Sigma-Aldrich, St. Louis, MO, USA) and incubated for 1 h at 37 ℃. Released sporozoites were collected by centrifugation at 13,200 × *g* for 3 min and washed three times with PBS.

### HCT-8 cell culture

Human colon adenocarcinoma cells (HCT-8 cells) were purchased from the Shanghai Branch of the Chinese Academy of Sciences and cultured in the Roswell Park Memorial Institute (RPMI)-1640 medium supplemented with 10% heat-inactivated fetal bovine serum (Gibco), 100 U/mL penicillin, and 100 μg/mL streptomycin at 37 ℃ under 5% CO_2_.

### Phylogenetic analysis

The amino acid sequences of the *NFDQ* genes in *C*. *parvum*, *C*. *hominis*,*C*. *bovis*, and *C*. * ryanae* were obtained from the National Center for Biotechnology Information (NCBI) website (www.ncbi.nlm.nih.gov). The NFDQ sequences were aligned with MUSCLE program implemented in MEGA v10 and phylogenetic trees based on maximum likelihood were constructed with 1000 replications for bootstrapping.

### Primers

All PCR primers used in the study were synthesized by Tsingke Biotech (Beijing, China) and are listed in Additional file 1: Table S1.

### Construction of plasmids and transgenic parasite strains

CRISPR-associated protein 9 (Cas9)/U6 plasmid was used for all genomic modification of the parasite [25]. To construct the Cas9/U6 plasmid for gene tagging and knockout, the eukaryotic pathogen clustered regularly interspaced short palindromic repeats (CRISPR) guide RNA/DNA design tool (http://grna.ctegd.uga.edu) was used to design a guide RNA targeting the *NFDQ1*. A guide RNA targeting the middle part of the *NFDQ1* was cloned into the Cas9/U6 plasmid. To construct homology repair template plasmids for gene tagging, the 800–900 bp segment around the stop codon of the *NFDQ1* coding region was used as homologous arms and the sequences were inserted into pNFDQ1-3HA-Nluc-P2A-neo plasmid by Gibson assembly cloning. To construct the gene deletion plasmid, the 5′ UTR and 3′ UTR genomic sequences of *NFDQ1* were used as homologous arms with specific primers, and the sequences were inserted into NFDQ1-Nluc-P2A-neo-NFDQ1 plasmid by Gibson assembly cloning. The PCR primers used are listed in Additional file 1: Table S1.

To obtain the NFDQ1-HA strain, 4 × 10^7^ excysted sporozoites were suspended in SF buffer (Lonza, Basel, Switzerland) and then co-electroporated with 50 μg pNFDQ1-3HA-Nluc-P2A-neo plasmid and 50 μg pACTIN::Cas9-U6::sg-NFDQ1 plasmid using the AMAXA 4D nucleofector system (Lonza) with the program of EH100. To obtain the *ΔNFDQ1* strain, 4 × 10^7^ excysted sporozoites were co-electroporated with 50 μg NFDQ1-Nluc-P2A-neo-NFDQ1 plasmid and 50 μg pACTIN::Cas9-U6::sg-NFDQ1 plasmid under the same conditions described above. A GKO mouse was orally gavaged with 200 μL of 8% (wt/vol) sodium bicarbonate 5 min before infection. Then, transfected parasites were immediately gavaged into GKO mice. For amplification and selection of transgenic strains, GKO mice were treated with 16 g/L paromomycin (Yuanye, Shanghai, China) drinking water from 24 h post-transfection throughout experiment. Fecal pellets were collected from 5 days post-infection and stored at 4℃ until further processing.

### Genotypic analysis of transgenic parasites by PCR

Genomic DNA was extracted from approximately 100 mg of feces using the Fast DNA SPIN Kit for Soil (MP Biomedicals, CA, USA). The PCR reaction was performed using genomic DNA template, Phanta Max Super-Fidelity DNA Polymerase (Vazyme, Nanjing, China) and the primers listed in Additional file 1: Table S1. Then, PCR products were analyzed by electrophoresis and imaged on the GelDoc XR^+^ system (Bio-Rad, Hercules, CO, USA).

### Luciferase assay

The Nano-Glo Luciferase assay kit (Promega, Madison, WI, USA) was used to perform the luciferase assay as previously described [26]. A fecal pellet was transferred to a 1.5 mL microcentrifuge tube containing ten 3 mm glass beads (Thermo Fisher Scientific, Waltham, MA, USA) and 1 mL fecal lysis buffer containing 2 mM DTT, 2 mM EDTA pH 8.0, 50 mM Tris pH 7.6, 1% Triton X-100, and 10% glycerol in ddH_2_O. After homogenization for 1 min, the supernatant was collected by centrifugation at 19,000 × *g* for 1 min. The 50 μL supernatant was combined with 50 μL of a 25:1 Nano-Glo Luciferase Buffer to Nano-Glo Luciferase Substrate Mix in a white 96-well plate (Sangon, Shanghai, China). After incubation for 3 min, the luminescence was measured by Synergy HTX imaging multimode reader (BioTek, Winooski, VT, USA).

### Western blot analysis

HCT-8 cells were plated in six-well culture plates and grown to confluence. One well of cells was infected with 2 × 10^7^ released sporozoites and cultured for 24 h. The samples were then lysed with RIPA lysis buffer (Beyotime, Shanghai, China), mixed with 5 × protein loading buffer (Yeasen, Shanghai, China), and boiled for 10 min. Lysed proteins were separated by sodium dodecyl sulfate–polyacrylamide gel electrophoresis (SDS-PAGE), transferred to a PVDF membrane (GE, Boston, MA, USA) and blocked with 5% skimmed milk-TBST for 2 h. After three washes, the membranes were incubated with rabbit anti-HA antibody (CST, Danvers, MA, USA) diluted 1: 800 for 1 h followed by HRP-conjugated goat anti-rabbit immunoglobulin G (IgG) (Beyotime, Shanghai, China) diluted 1:1000 as secondary antibody for 1 h at RT. Finally, the specific bands on the membrane were detected by the ECL method (Beyotime, Shanghai, China), measured on the Chemstudio imaging system (Analytik Jena, Germany).

### Indirect Immunofluorescence Assay (IFA)

To determine the localization of NFDQ1 in each developmental stage of *C*. *parvum*, sporozoites were used to infect fresh HCT-8 cells on coverslips and cultured for 24 or 48 h prior to staining. All samples were incubated with 4% paraformaldehyde (Leagene, Beijing, China) for 15 min, permeabilized with 0.3% Triton X-100 (Sigma-Aldrich), for 15 min, and blocked with 1% BSA (Biofroxx, Guangzhou, China) for 1 h. Rabbit anti-HA antibodies were diluted 1: 800 in PBS for 1 h at room temperature and washed with PBS. Secondary antibodies were diluted in PBS for staining: secondary antibodies conjugated with Alexa Fluor dyes (Thermo Fisher Scientific) at 1:1000, Sporo-Glo (Waterborne, New Orleans, LA, USA) was used at 1:50, *Vicia villosa* lectin fluorescein (Vector Laboratories, Newark, CA, USA) was used at 1:400. Cells or sporozoites were incubated with secondary antibodies for 1 h at room temperature and the nucleus was stained with Hoechst (Thermo Fisher Scientific) diluted 1:2000 in PBS for 10 min at room temperature. After five washes with PBS, the cultures were imaged using a BX53 fluorescence microscope (Olympus, Tokyo, Japan). Images were analyzed using ZEN 2 (blue edition) software.

### Immunoelectron microscopy

A GKO mouse was infected with 1 × 10^3^ NFDQ1-HA transgenic oocysts and the ileum was harvested 13 days after infection. The ileum was sectioned, washed three times with cold PBS and fixed in freshly prepared 4% paraformaldehyde and 0.1% glutaraldehyde (Ted Pella, Redding, CA, USA) mixture for 4 h at 4 °C. Following gradient dehydration, the samples were infiltrated in LR-White resin (Sigma-Aldrich) for overnight, then transferred to a fresh LR-White resin for the further infiltration at −20 °C for 24 h. Samples were polymerized, trimmed, and sectioned using a Leica EM UC7 ultramicrotome (Wetzlar, Germany). Ultrathin 70 nm sections were blocked with 1% BSA for 20 min and then incubated with rabbit anti-HA antibody (CST, Danvers, MA, USA) overnight at 4 °C. After washing with PBS containing 0.1% BSA, the sections were incubated with 10 nm colloidal gold conjugated goat anti-rabbit IgG (Sigma-Aldrich) at 37 °C for 60 min. Finally, the sections were stained with 2% uranyl acetate (w/v) (SPI, West Chester, PA, USA) and viewed on a Talos L120C transmission electron microscope (Thermo Fisher Scientific).

### In vitro growth assay of *C*. *parvum*

HCT-8 cells were plated in 48-well culture plates and grown until confluent. One well of cells was infected with 1 × 10^4^ transgenic NFDQ1-HA or *ΔNFDQ1* oocysts per well. At 3, 12, 24, 36, or 48 h post infection (pi), monolayers were fixed with 100 μL Nano-Glo Luciferase Buffer at 37 °C for 10 min. Total samples were transferred to a white 96-well plate and 2 μL Nano-Glo Luciferase Substrate was added. The plate was incubated for 3 min in the dark at room temperature and the nanoluciferase was measured using a Synergy HTX imaging multimode reader (BioTek).

### Statistical analysis

Statistical analyses were performed with GraphPad Prism 8.0. Information on the specific statistical tests and *P* values are provided in the legends for each fig. Differences with *P* < 0.05 were considered significant.

## Results

### *NFDQ1-NFDQ3* have distinct gene expression profiles

**Fig. 1:**
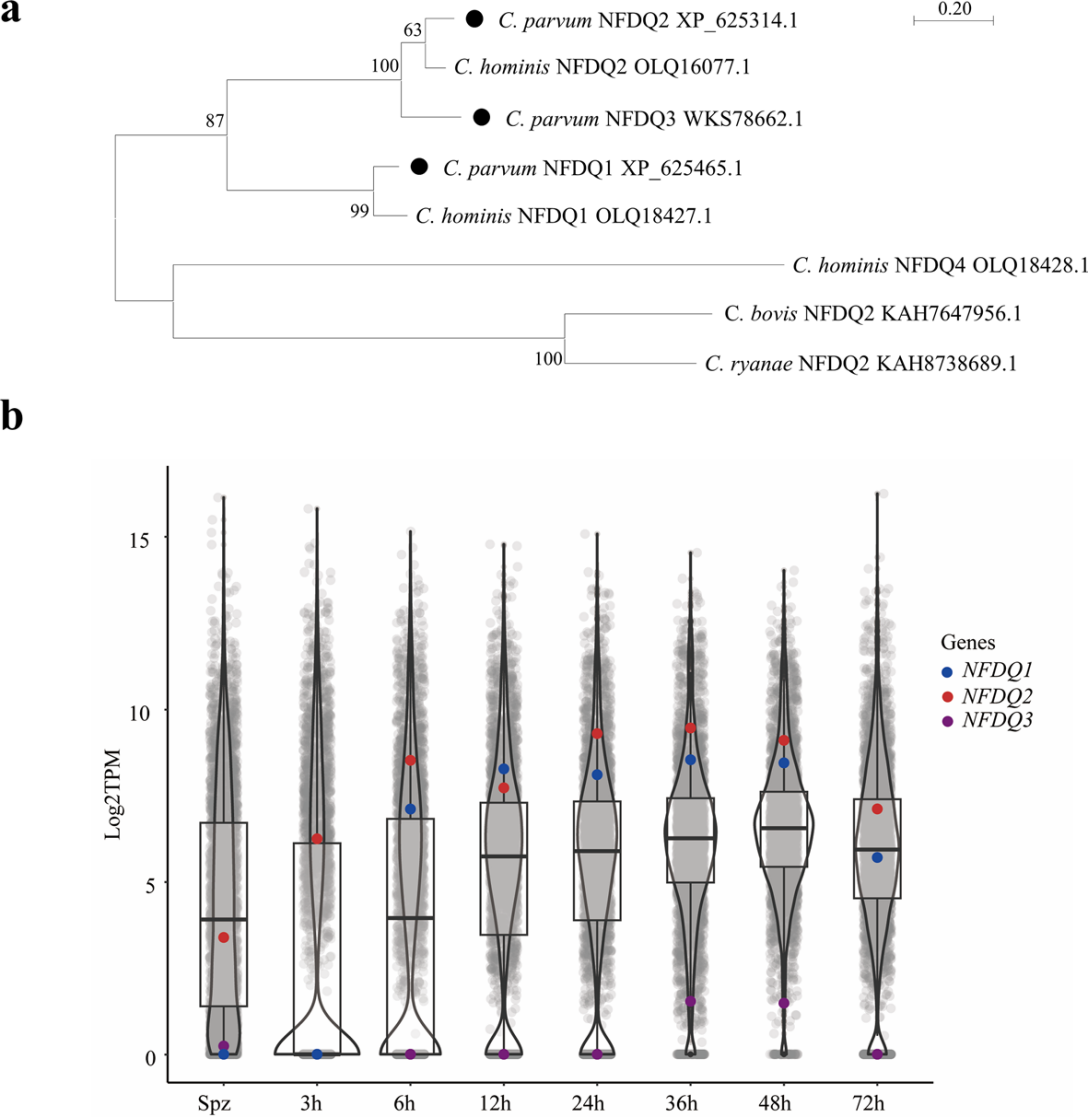
*Cryptosporidium parvum* contains three *NFDQ* genes. **a** Phylogenetic relationship of NFDQ among *C*. *parvum*, *C*. *hominis*, *C*. *bovis*, and *C*. *ryanae*. The tree was constructed by a maximum likelihood method and Jones–Taylor–Thornton (JTT) model with 1000 replications for bootstrapping. Scale bar = 0.2 of substitutions per amino acid site. **b** Violin plots demonstrate the relative expression of all *Cryptosporidium* genes (arranged by chromosome) in sporozoites and developing stages in HCT-8 cells after infected with the IIdA20G1-HLJ isolate of *C*. *parvum*, as indicated by TPM values from RNA-seq analysis of the transcriptome. Each dot represents one gene, with the expression of three *NFDQ* genes being indicated (*n* = 4)

Three *NFDQ* genes, cgd8_10 (referred to here as *NFDQ1*), cgd6_5500 (referred to here as *NFDQ2*), and cgd6g5500 (referred to here as *NFDQ3*), were previously identified in the genome of *C*. *parvum*. To better understand their relationships, we compared the protein sequences of NFDQs among *C*. *parvum*, *C*. *hominis*, *C*. *bovis*, and *C*. *ryanae*. The phylogenetic analysis indicated that the NFDQ from *C*. *parvum* is closely related to that of *C*. *hominis*, but more distantly related to those from *C*. *bovis* and *C*. *ryanae* (Fig. 1a). The evolutionary relationship of cgd6_5500 is closer to cgd6g5500 than to cgd8_10 (Fig. 1a). Furthermore, cgd8_10 is unique to *C*. *parvum* and *C*. *hominis* and has no homologs in *C*. *bovis* and *C*. *ryanae*. Transcriptomic analysis revealed that the three *NFDQ* genes are differentially expressed in sporozoites and have distinct expression patterns in intracellular developmental stages. In particular, *NFDQ3* is specifically expressed during sexual reproduction, whereas others are expressed during both asexual and sexual reproduction. Among the latter, *NFDQ1* is not expressed during early development (3 h) (Fig. 1b).

### Epitope tagging of NFDQ1 in *Cryptosporidium parvum*

**Fig. 2:**
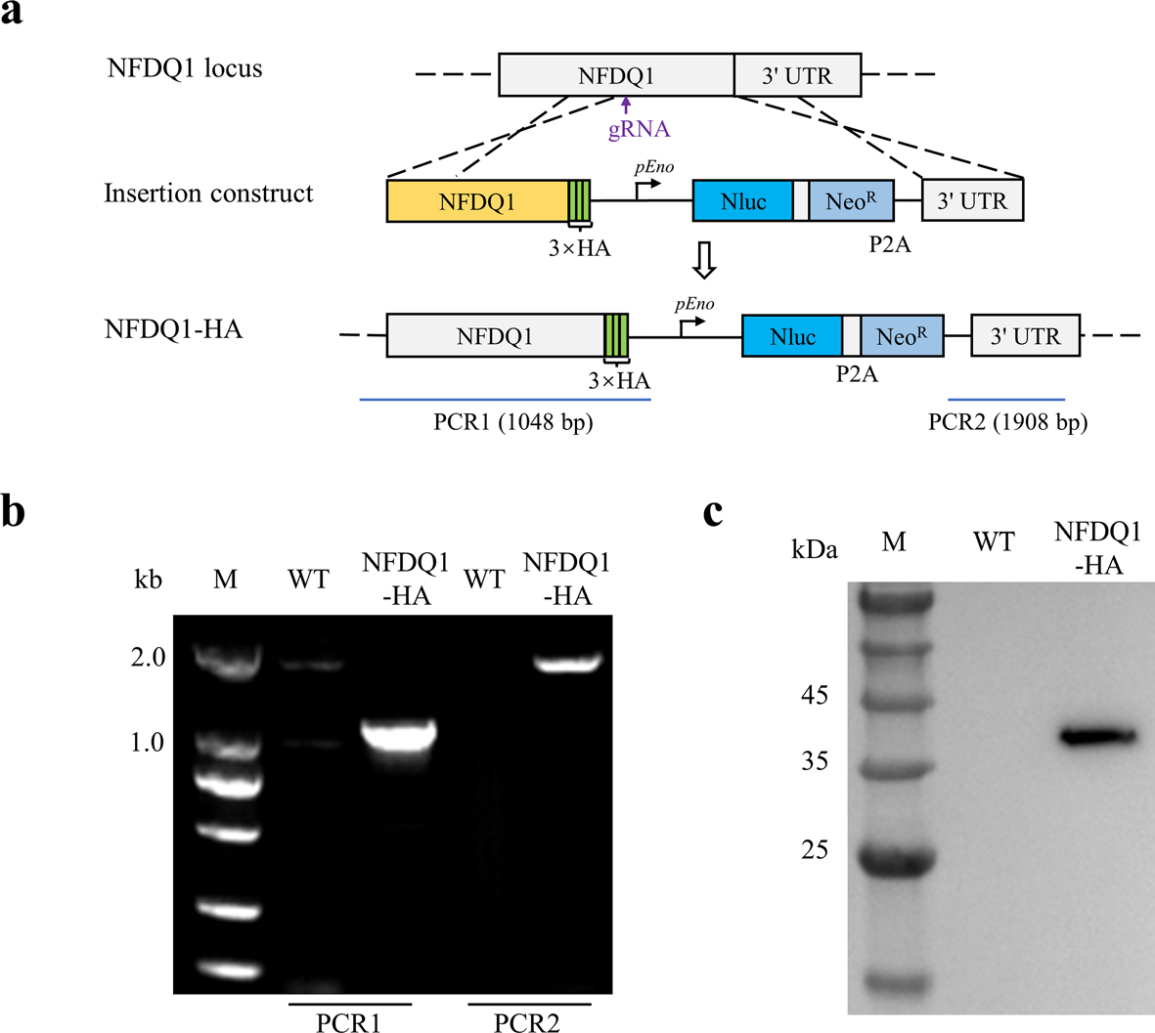
Genetic tagging of NFDQ1 of *Cryptosporidium parvum*. **a** Diagrams of endogenous gene tagging for NFDQ1. The C-terminus of NFDQ1 gene has been replaced with a repair template that includes a 3 × hemagglutinin (HA) epitope, a nanoluciferase reporter gene (Nluc), and a neomycin resistance gene (Neo^R^). **b** Diagnostic PCR of the NFDQ1-HA locus. Fecal genomic DNA from wild-type (WT) or NFDQ1-HA parasites was used as template, and primers for checking 5′ insertion (PCR1) and 3′ insertion (PCR2) are indicated. **c** Western blot analysis of the expression of NFDQ1-HA. HCT-8 cells were infected with NFDQ1-HA or WT oocysts. After 24 h post-infection (hpi), cells were lysed and detected by rabbit anti-HA monoclonal antibody

To access the expression and localization of NFDQ1 in *C*. *parvum*, we introduced three hemagglutinin epitopes (3 × HA) at the C-terminus of NFDQ1, and the NanoLuc luciferase (Nluc) and neomycin resistance gene (Neo^R^) driven by an enolase promoter at the downstream of *NFDQ1* using CRISPR/Cas9 technology (Fig. 2a). After selection in the GKO mouse, the NFDQ1-HA transgenic strain was obtained. The correct 5′ and 3′ integration for the tagged *NFDQ1* locus was identified by diagnostic PCR, with the expected bands of PCR1 and PCR2 analyses being present in the NFDQ1-HA strain, but absent in the wild-type (WT) strain (Fig. 2b). To determine the expression of NFDQ1, HCT-8 cells were infected with NFDQ1-HA or WT strain, and the culture was harvested and lysed at 24 hpi for western blotting. The result showed that the ~ 36 kDa band was detected in the NFDQ1-HA sample but not in the WT sample using an anti-HA monoclonal antibody (Fig. 2c). These results demonstrate that the 3HA tag is correctly inserted into the *NFDQ1* locus and that NFDQ1 is expressed in the parasites after 24 h of culture in vitro.

### NFDQ1 expressed in cytoplasm of parasites

**Fig. 3:**
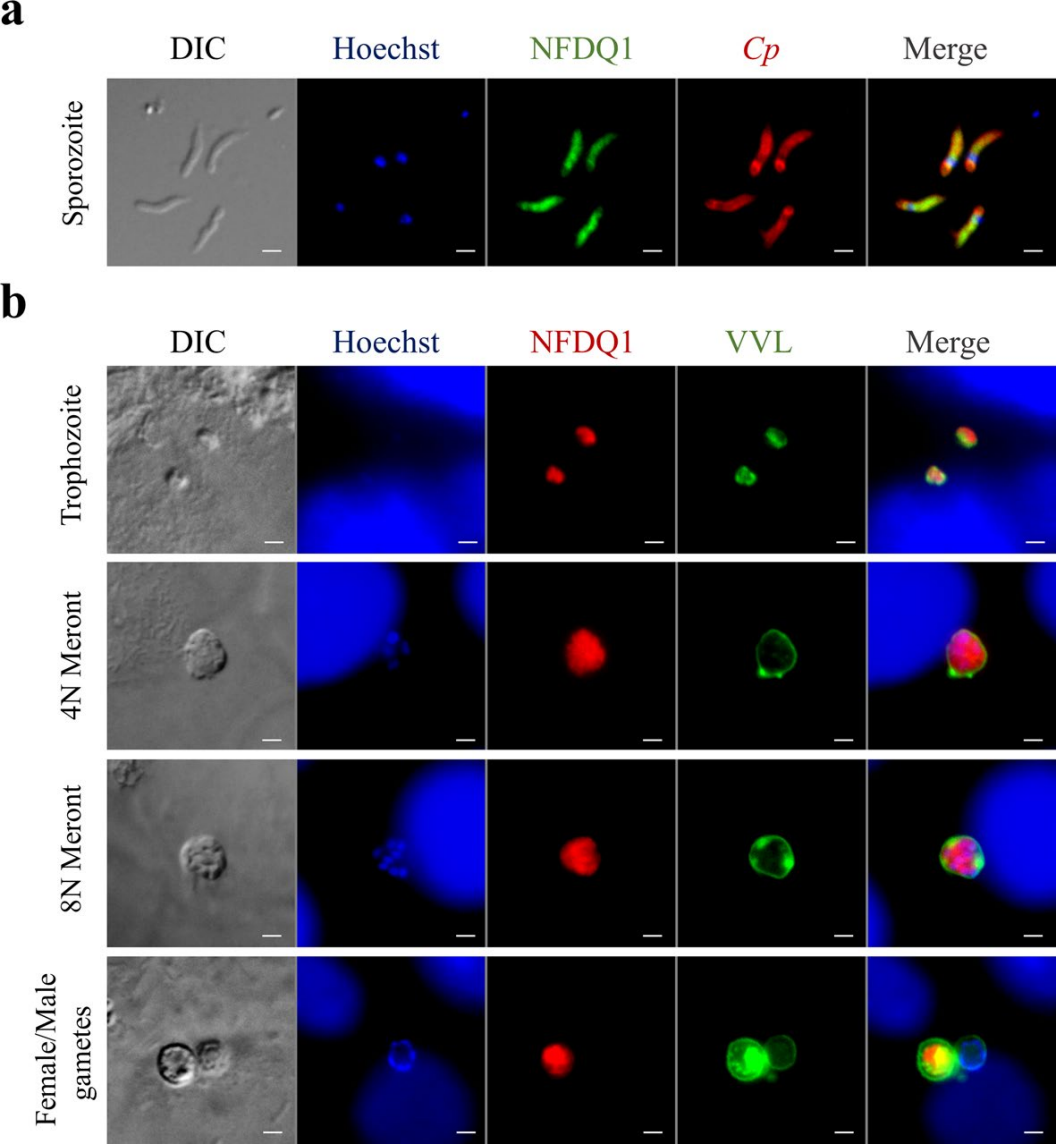
Expression of NFDQ1 in *C*. *parvum* life cycle stages indicated by immunofluorescence. **a** Immunofluorescence analysis of NFDQ1-HA in sporozoites. NFDQ1 sporozoites were fixed and stained with rabbit anti-HA mAb (green), Sporo-Glo (*Cp*, red), and Hoechst (blue). Scale bars = 2 µm. **b** Immunofluorescence analysis of NFDQ1-HA in intracellular parasites. HCT-8 cells were infected with NFDQ1-HA oocysts, fixed at 24 hpi or 48 hpi, and stained with rabbit anti-HA mAb (red), VVL (green), and Hoechst (blue). Scale bars = 2 µm

To study the localization of NFDQ1 in the parasite, HCT-8 cells were infected with NFDQ1-HA transgenic parasites and cultures were fixed at 24 or 48 hpi for immunofluorescence assay. In addition, free NFDQ1-HA sporozoites were used for immunofluorescence assay to determine the expression of NFDQ1 in the extracellular stage of the parasite. In free sporozoites, NFDQ1 was colocalized with Sporo-Glo which is a *C*. *parvum* maker and staining the entire of the sporozoites (Fig. 3a). In the intracellular developmental stages, the mAb against the HA tag reacted to trophozoites and meronts with similar staining patterns. In addition, the fluorescent signal did not colocalize with *Vicia villosa* lectin (VVL), which stained the parasitophorous vacuole (PV), whereas the anti-HA antibody stained almost the entire parasite. In the sexual stage, the anti-HA antibody reacted strongly with the entire female gametes, with no reactivity with male gametes (Fig. 3b). These results indicate that NFDQ1 is localized to the cytoplasm of *C*. *parvum* in almost all life stages of the parasites, except the male gametes.

**Fig. 4:**
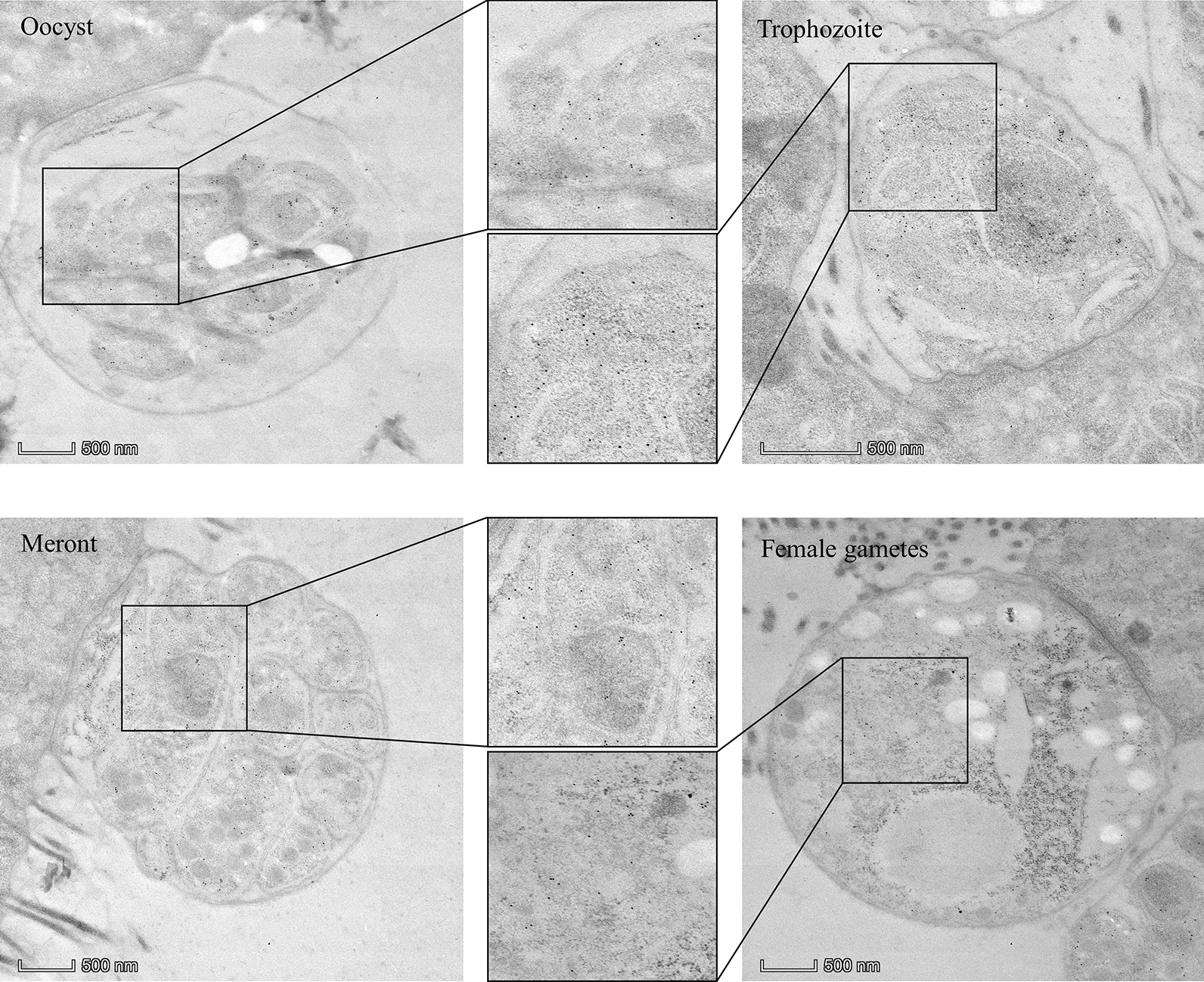
Immunoelectron microscopy micrographs of NFDQ1-HA parasites. A GKO mouse was infected with NFDQ1-HA oocysts. After 13 days after infection, the ileum was fixed and stained with rabbit anti-HA antibody followed by 10 nm colloidal gold conjugated goat anti-rabbit IgG. Scale bars = 500 nm

To further study the ultrastructural localization of NFDQ1, NFDQ1-HA transgenic parasites were infected with a GKO mouse, and the mouse ileum was collected and examined by immunoelectron microscopy. The results revealed that the anti-HA gold particles were detected in the entire sporozoites, trophozoites, meronts and female gametes (Fig. 4). In sporozoites, gold particles were mainly found in the cytoplasm, while no gold particles were found in secretory organelles and on the surface of sporozoites. In trophozoites and meronts, gold particles were distributed in large amounts in the cytoplasm and a few particles were found in the nucleus. In female gametes, the particles were also distributed in the cytoplasm. These images are consistent with the immunofluorescence analysis of NFDQ1 as shown above, suggesting that NFDQ1 is a cytoplasmic protein in *C*. *parvum*.

### Ablation of *NFDQ1* in *C*. *parvum* using CRISPR/Cas9 system

**Fig. 5:**
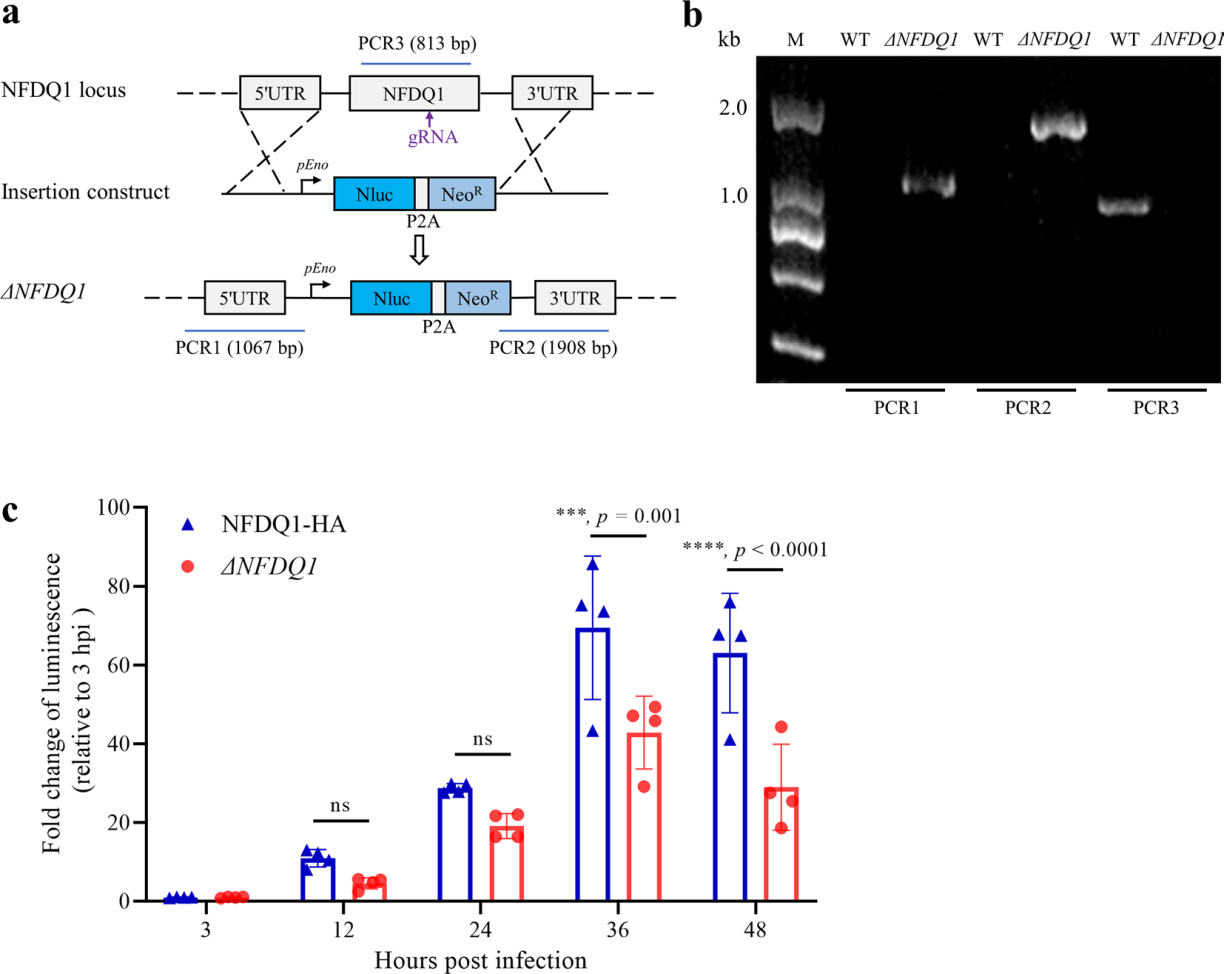
Influences of *ΔNFDQ1* parasites on growth of *C*. *parvum*in vitro. **a** Diagram of the strategy to construct *ΔNFDQ1* transgenic strain. The construct was designed to replace the NFDQ1 locus with a Nluc-P2A-Neo^R^ cassette. **b** Diagnostic PCR of the *ΔNFDQ1* locus. Fecal genomic DNA from WT or *ΔNFDQ1* parasites was used as template, and primers for checking 5′ insertion (PCR1), 3′ insertion (PCR2), and endogenous NFDQ1 locus (PCR3) are indicated. **c** Luciferase fold changes between growth of NFDQ1-HA and *ΔNFDQ1* parasites in vitro. HCT-8 cells were infected with NFDQ1-HA or *ΔNFDQ1* oocysts. After specific time points, parasite growth was measured by nano-luciferase assay. *n* = 3 independent experiments, means ± SD; *P* = exact values or ns (not significant) shown as determined with two-way ANOVA corrected for multiple comparisons according to Sidak’s method

To further investigate the function of NFDQ1 in *C*. *parvum*, we generated a knockout mutant of *NFDQ1* (*ΔNFDQ1*) strain. The *NFDQ1* gene was replaced with the Nluc and Neo^R^ driven by an enolase promoter (Fig. 5a). The *ΔNFDQ1* strain was easily obtained from GKO mice with the paromomycin selection. Correct genomic integration was confirmed by the presence of the expected bands from PCR1 and PCR2 analyses in the *ΔNFDQ1* strain but not in the WT strain. Furthermore, primers for the open reading frame of NFDQ1 failed to amplify products from the knockout strain, although they readily detected the expected fragment from WT parasites (Fig. 5b).

### Loss *NFDQ1* reduces parasite growth in vitro

To investigate the function of NFDQ1 in the parasite life cycle, we utilized the luciferase-based growth assay to compare the growth of WT parasites with *ΔNFDQ1* transgenic parasites. HCT-8 cells were infected with 10^4^ oocysts per well for each strain. After specified times, cells were lysed and incubated with Nluc buffer containing substrate to detect luminescence. The results showed that the intracellular growth ability of *ΔNFDQ1* was significantly different from that of the WT strains, especially during sexual replication in vitro (Fig. 5c). The luminescence levels of cells infected with the *ΔNFDQ1* strain were significantly lower than those of cells infected with the WT strain at 36 hpi (Fig. 5c). These results indicate that NFDQ1 is required for parasite growth in vitro.

## Discussion

The *Cryptosporidium parvum* genome encodes three *NFDQ* genes located in the subtelomeric region, but the functions of NFDQs have not been studied. In this study, we employed CRISPR/Cas9 technology to tag *NFDQ1* gene at endogenous loci. NFDQ1 was expressed in both asexual and sexual stages of *C*. *parvum* and localized to the cytoplasm of the parasite. Deletion of *NFDQ1* resulted in an apparent growth retardation, particularly during sexual replication in vitro. Our studies suggest that NFDQ1, as a cytoplasmic protein, is likely involved in parasite growth in vitro.

The function of the NFDQ proteins remains unclear. The NFDQ family, identified by comparative genomic analysis of three common intestinal *Cryptosporidium* species in cattle, shows significant sequence similarity among its members. Each member has a conserved amino acid sequence beginning with NF and ending with DQ. The biological importance of the NFDQ proteins is reflected in their number. *C*. *parvum* contains three genes encoding NFDQ proteins, whereas *C*. *bovis* and *C*. *ryanae* each have only one gene each. Comparative genomic analysis suggests a potential role for NFDQ proteins in host specificity and pathogenicity [18]. This hypothesis is supported by the fact that *C*. *parvum*, which causes moderate to severe diarrhea or even death, has three *NFDQ* genes, compared with the single *NFDQ* gene in *C*. *bovis* and *C*. *ryanae*, species that primarily cause subclinical infections [27, 28].

NFDQ1 is expressed in the cytoplasm of the parasite during all life cycle stages except in male gametes. Immunofluorescence analysis using a monoclonal antibody to the HA tag showed strong reactivity with sporozoites, trophozoites, meronts, and female gametes, but no reactivity with male gametes, and the staining patterns were consistent throughout the parasite. Furthermore, NFDQ1 was not localized to any secretory organelles in our study and was not found in the secreted effector proteome [29], suggesting that NFDQ1 is a cytoplasmic protein without secreting characters during parasite development. Members of the multigene families, such as MEDLE, ROP, and mucin in *Cryptosporidium*, show diverse localization and some proteins are transported into host cells. Endogenous tagging of the MEDLE family revealed that MEDLE1, MEDLE2, and MEDLE6 are secreted into the host cell cytoplasm, with MEDLE2 possessing a host-targeting sequence similar to some secretory proteins in *Plasmodium *spp. and *Toxoplasma gondii* [30]. Similarly, endogenous tagging of potential rhoptry proteins (ROPs) in *C*. *parvum* identified six ROP proteins, with ROP1 and ROP3 found both in the parasitophorous vacuole (PV) and intracellularly [31]. Therefore, future studies on NFDQ2 and NFDQ3 could determine whether NFDQ proteins are transported into host cells to modulate host functions.

NFDQ1 may participate in parasite growth in vitro. As a highly polymorphic gene, *NFDQ1* was predicted to potentially contribute to the host range and pathogenicity [18]. In this study, knocking out the *NFDQ1* gene affected the growth of *C*. *parvum* in HCT-8 cells. In addition, the *ΔNFDQ1* parasite showed a decreasing development compared to the NFDQ1 tagged parasites at 36 hpi and 48 hpi. To further determine the role of the NFDQ1 in parasite infectivity, in future, we could use immunocompromised mice infected with *ΔNFDQ1* strain to observe the parasite burden in vivo. In addition, members of the multigene families in *Cryptosporidium* have been demonstrated to be involved in the invasion of host cells and the regulation of host cellular processes. *C*. *parvum* ROP1 enters host cells during *C*. *parvum* invasion and interacts with LMO7 to affect cell polarity, and MEDLE2, which is exported into host cells after invasion, induces endoplasmic reticulum stress in mammalian cells [30, 31]. *NFDQs* belong to a multi-gene family in *C*. *parvum*, and future studies are needed to elucidate their function in *Cryptosporidium*.

## Conclusions

We used CRISPR/Cas9 technology to generate NFDQ1-HA tagged and NFDQ1 knockout (*ΔNFDQ1*) strains. Immunofluorescence and immunoelectron microscopy showed that NFDQ1 is highly expressed in the cytoplasm during both asexual and sexual stages of *C*. *parvum*, except in male gametes. Loss of *NFDQ1* impaired parasite growth in vitro, suggesting that NFDQ1 may be involved in parasite development. This study initially investigated the localization and function of NFDQ1 in *C*. *parvum* provides new insights into the development of effective therapies against the infection.

## Supplementary Information

Additional file 1: Table S1. Primers used in this study.

## Data Availability

No datasets were generated or analyzed during the current study.

## Abbreviations

CRISPR: Clustered regularly interspaced short palindromic repeats
Cas9: CRISPR-associated protein 9
SDS-PAGE: Sodium dodecyl sulphate–polyacrylamide gel electrophoresis
PVDF: Polyvinylidene fluoride
TBST: Tris-buffered saline with Tween-20
WT: Wild type
